# Kinetics of Fluorescein in Tear Film After Eye Drop Instillation in Beagle Dogs: Does Size Really Matter?

**DOI:** 10.3389/fvets.2019.00457

**Published:** 2019-12-19

**Authors:** Lionel Sebbag, Nicolette S. Kirner, Rachel A. Allbaugh, Alysha Reis, Jonathan P. Mochel

**Affiliations:** ^1^Department of Veterinary Clinical Sciences, College of Veterinary Medicine, Iowa State University, Ames, IA, United States; ^2^Department of Biomedical Sciences, SMART Pharmacology, College of Veterinary Medicine, Iowa State University, Ames, IA, United States; ^3^Lloyd Veterinary Medical Center, College of Veterinary Medicine, Iowa State University, Ames, IA, United States

**Keywords:** tear film, drainage, canine, tear flow, fluorophotometry, eye drop, drug delivery

## Abstract

The study aimed to determine the impact of drop size on tear film pharmacokinetics and assess important physiological parameters associated with ocular drug delivery in dogs. Two separate experiments were conducted in eight healthy Beagle dogs: (i) Instillation of one drop (35 μl) or two drops (70 μl) of 1% fluorescein solution in each eye followed by tear collections with capillary tubes from 0 to 180 min; (ii) Instillation of 10 to 100 μl of 0.1% fluorescein in each eye followed by external photography with blue excitation filter (to capture periocular spillage of fluorescein) and tear collections from 1 to 20 min (to capture tear turnover rate; TTR). Fluorescein concentrations were measured in tear samples with a fluorophotometer. The TTR was estimated based upon non-linear mixed-effects analysis of fluorescein decay curves. Tear film pharmacokinetics were not superior with instillation of two drops vs. one drop based on tear film concentrations, residual tear fluorescence, and area under the fluorescein-time curves (*P* ≥ 0.163). Reflex TTR varied from 20.2 to 30.5%/min and did not differ significantly (*P* = 0.935) among volumes instilled (10–100 μl). The volumetric capacity of the canine palpebral fissure (31.3 ± 8.9 μl) was positively correlated with the palpebral fissure length (*P* = 0.023). Excess solution was spilled over the periocular skin in a volume-dependent manner, predominantly in the lower eyelid, medial canthus and lateral canthus. In sum, a single drop is sufficient for topical administration in dogs. Any excess is lost predominantly by spillage over the periocular skin as well as accelerated nasolacrimal drainage.

## Introduction

Topical administration is the route of choice for treating diseases that affect the anterior segment of the eye (1, 2). This route is simple, convenient, non-invasive, and allows for the use of relatively high drug concentrations at the target tissue while minimizing systemic exposure (1, 2). One of the main challenges associated with topical administration, however, remains the poor bioavailability of therapeutic drugs to the inner tissues of the eye given rapid precorneal loss from reflex blinking and efficient nasolacrimal drainage (3–5). Optimization of eyedrop delivery can enhance therapeutic benefits for the patient (2, 6), regardless of the underlying pathology (e.g., dry eye, infectious keratitis, glaucoma), yet little consensus exists on fundamental concepts such as the number of eyedrops to apply. The label of ophthalmic products often recommends to “apply one to two drops” (e.g., Optixcare®, Lotemax®), while diverse publications in veterinary and human ophthalmology describe the use of either “1 drop” (7, 8), “1 to 2 drops” (9, 10), or “2 drops” (11, 12).

The volume of solution instilled through topical administration is known to influence the precorneal residence time, a key parameter in ocular pharmacology (5, 13). A prolonged contact time between the solution and the ocular surface is often desired, as it enhances drug bioavailability and permits longer intervals between instillations (6). In humans, best practices for ocular delivery often recommend a single drop of commercial preparations (~35 μl) per dosing session (2, 5, 6), as the maximum volume that the human palpebral fissure can hold without overflowing is estimated at 25–30 μl (1, 14). Any excess is rapidly lost *via* nasolacrimal drainage and spillage over the eyelashes and periocular skin (6, 15); therefore, a second drop does not provide any therapeutic advantage in humans and may in fact be counterproductive by increasing systemic absorption and the risk of associated adverse effects. In rabbits, a single drop is also sufficient as the lacrimal drainage rate is proportional to the volume of solution instilled (up to 50 μl), hence tear film drug concentrations decrease less rapidly with lower instilled volumes (13, 16). In fact, the smaller the instilled volume, the greater the fraction of applied dose that is absorbed inside the rabbit's eye (13, 16). Similar findings may be true in dogs, albeit direct extrapolation between species is not possible given important differences in ocular anatomy and physiology. In particular, the canine tear volume (65.3 μl) (17) is nearly 9-fold larger than humans (7.0 μl) (14) and rabbits (7.5 μl) (13), while the canine tear turnover rate is comparable to humans (12.2%/min vs. 10–20%/min, respectively) (17, 18) but faster than rabbits (7.1%/min) (13).

The primary objective of this study was to determine the influence of volume instilled *via* topical administration (i.e., one vs. two drops) on tear film kinetics of fluorescein in dogs. Given the aforementioned species differences in tear film dynamics, we originally hypothesized that the kinetic profile would be superior following instillation of two drops in canine eyes, a hypothesis that was proven to be wrong. Hence, to explain why a single eyedrop is deemed sufficient in dogs, a secondary objective was to determine the maximal volume that the canine palpebral fissure can hold, as well as the drainage rate relative to diverse volumes (10–100 μl) instilled onto the canine ocular surface. The present work focuses on canine-specific ocular physiology, providing valuable information to veterinary practitioners, pet owners, and scientists working with dogs as a translational animal model for ocular surface diseases.

## Materials and Methods

### Animals

Eight Beagle dogs (four neutered male, four spayed female) were included in the study, all confirmed to be healthy based on physical and ophthalmic examinations performed by a board-certified veterinary ophthalmologist (LS), including Schirmer tear test-1 (Eye Care Product Manufacturing, LLC, Tucson, AZ, USA), rebound tonometry (TonoVet, Icare Finland Oy, Espoo, Finland), slit-lamp biomicroscopy (SL-17; Kowa Company, Ltd., Tokyo, Japan), and indirect ophthalmoscopy (Keeler Vantage; Keeler Instruments, Inc., Broomall, PA, USA). All dogs were 3.0–3.5 years old and weighed 7.5–10 kg. The study was approved by the Institutional Animal Care and Use Committee of Iowa State University (IACUC #18-398) and was conducted in accordance with the Association for Research in Vision and Ophthalmology statement for the use of animals in ophthalmic and vision research.

### Tear Film Fluorescein Following Instillation of One vs. Two Drops

A 1% fluorescein concentration was obtained by mixing 10% fluorescein solution (Akorn Inc., Lake Forest, IL, USA) with 1.4% polyvinyl alcohol lubricating eye drops (Artificial Tears, Rugby, Rockville Center, NY, USA). On Day 1, one eye in each dog was randomly selected (Excel software, Microsoft Corp., Redmond, WA, USA) to receive 35 μl (one drop) of 1% fluorescein solution while the contralateral eye received 70 μl (two drops) of the same solution, using a pipette (Eppendorf Reference® 2, 10–100 μl) for accuracy. On Day 2 (24 h later), the order of eyes was reversed and the experiment was repeated. Of note, the volume chosen for a single drop (35 μl) approximates the average drop size of commercial ophthalmic preparations used in veterinary and human medicine (35–39 μl) (19, 20) and is routinely described in previous scientific publications (4, 8, 21, 22). Following topical instillation, tear fluid was collected in each eye with a 2-μl capillary glass tube (Drummond Scientific Co., Broomhall, PA, USA) at the following time points: 0 min (i.e., immediately after instillation and spontaneous blinking), 1, 5, 10, 20, 30, 40, 50, 60, 90, 120, and 180 min. The capillary tube was placed against the inferior tear lake for ≤2 s, a duration sufficient to collect tear fluid by capillary action while minimizing the risk of inadvertent ocular irritation and reflex tearing. Given the rapid collection time (<2 s), the lack of blinking during collection (eyelids manually opened), and the relatively large tear volume in dogs (~65 μl) (17), the authors believe that it is unlikely for reflex tearing, if any, to affect tear fluorescein concentrations in a significant manner. The length of fluid contained within each capillary tube was measured to the nearest millimeter using a ruler, a value used to calculate the volume of fluid collected (as 32 mm equates to 2 μl). The collected fluid was then expelled into a 2-ml Eppendorf tube that contained 500 μl of phosphate buffered saline (Gibco® PBS, pH 7.2, Thermo Fisher Scientific, Rockford, IL, USA), vortexed for 30 s, and transferred to a cuvette for analysis. Fluorescein concentrations were measured (in ng/ml) with a computerized scanning ocular fluorophotometer (Fluorotron Master^TM^, Coherent Radiation, Palo Alto, CA, USA) as previously described (17), with the exception that tear fluid was diluted with 0.5 ml of PBS herein (instead of 2 ml) to improve the sensitivity of fluorescein detection (data not shown); the cuvette was slightly raised in the device's cuvette holder to account for the lower total volume.

### Volumetric Capacity of the Palpebral Fissure

A 0.1% fluorescein concentration was obtained by mixing 10% fluorescein solution with 1.4% polyvinyl alcohol lubricating eye drops. Each eye received the following volumes of 0.1% fluorescein solution *via* pipette administration, the order being selected at random (Excel software) in each dog: 10, 20, 30, 40, 50, 60, 70, 80, 90, and 100 μl. To minimize any carry-over effect from one session to another, the eyes and periocular skin were thoroughly rinsed with eye wash (Ocusoft® Eye Wash, OcuSOFT Inc., Richmond, TX, USA) at completion of each experiment, and a 1-h break was provided between repeated instillations in each eye to allow ample time for the physiological tear film dynamics to be restored (17). At each session, within 10 s of topical instillation and spontaneous blinking, an external photograph was taken with a Nikon D90 camera to capture each eye and associated periocular skin. To enhance detection of fluorescence, the camera was equipped with a screw-on Tiffen Wratten 15 deep yellow filter (Tiffen Manufacturing, Hauppauge, NY, USA) as well as an external flash (Nikon Speedlight SB-700) covered with a blue excitation filter (SJ-4 blue color). Of note, this photographic method better highlighted 0.1% than 1% fluorescein, hence the choice of 0.1% solution for this experiment.

### Tear Turnover Rate at Various Instilled Volumes

In the experiment described above, following external photography (taken ~10 s after 0.1% fluorescein instillation), tear fluid was collected with 2-μl capillary glass tubes at the following time points in each eye: 1, 2, 4, 6, 10, 15, and 20 min (17). Tear film fluorescein concentrations were measured in all samples (see above for details) and recorded in ng/ml.

### Data Analysis

#### Fluorophotometry

First, a fluorescein calibration curve was established by analyzing a dilution series of known fluorescein concentrations in triplicate (1–10,000 ng/ml). Fluorescein concentrations in tear samples were corrected based on the resulting calibration equation (y = 19.3 + 0.9 x – 3E-05 x^2^) (17). Fluorescein data of each animal were inputted to Monolix® version 2019R1 (Lixoft, Orsay, France), and tear turnover rate (TTR) was derived from a non-linear mixed effects model as previously described (17), assessing both reflex (rTTR) and basal (bTTR) tear turnover rates. Selected data points were censored in Monolix when a peak of fluorescence could not be identified on the fluorophotometer reading, or if the tear fluorescein concentration did not make physiologic sense (e.g., higher fluorescein at 2 min compared with baseline) (17). Overall, 11/640 (1.7%) of all data points were left censored.

#### External Photography

**Figure 1:**
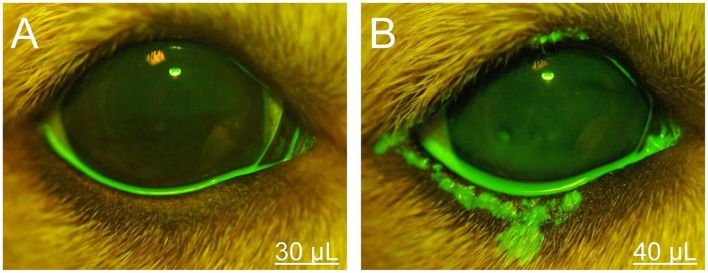
Photographs of the right eye and eyelids in a representative Beagle dog. The volumetric capacity of the palpebral fissure was calculated as 35 μl in this eye, based on the lack or presence of periocular spillage of 0.1% fluorescein with instillation of either 30 μl **(A)** or 40 μl **(B)**, respectively.

**Figure 2:**
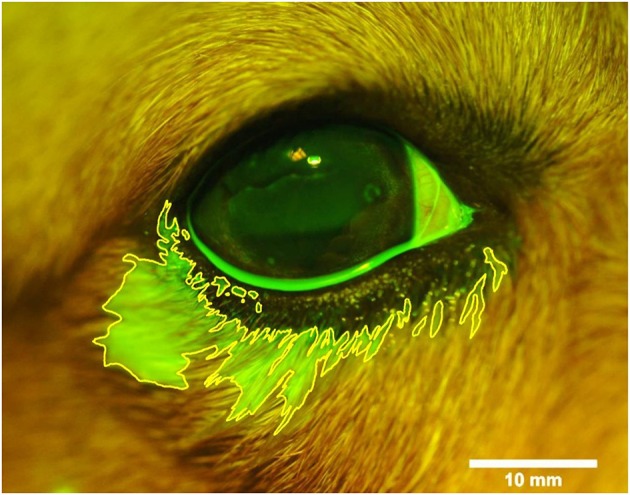
Photograph of the right eye and eyelids following topical instillation of 90 μl of 0.1% fluorescein in a representative Beagle dog. The area of periocular spillage was delineated with ImageJ software (version 1.52a, National Institute of Health), and recorded in mm^2^ based on a length bar (10 mm) specific to each eye.

The volumetric capacity of the palpebral fissure was calculated in each eye as the average between the lowest instilled volume that led to periocular spillage of fluorescein solution and the highest instilled volume for which all fluorescence remained on the ocular surface. For instance, a volumetric capacity of 35 μl was calculated for an eye that had spillage first noted at 40 μl of instilled solution, but no spillage was noted at 30 μl (Figure 1). When present, the location of spillage was recorded (i.e., lower eyelid, upper eyelid, medial canthus, lateral canthus), and the area of fluorescence that extended beyond the eyelids margins was delineated with the “freehand selection” tool in ImageJ 1.52a software (National Institutes of Health, Bethesda, MD, USA). The area of fluorescein spillage was recorded in mm^2^ (Figure 2) using a length bar specific to each eye (i.e., palpebral fissure length measured in mm with calipers).

#### Statistical Analysis

Normality of data was assessed with the Shapiro–Wilk test. A mixed model for repeated measures (MMRM) was fitted to the data using the R software version 3.6.0. In the model, fluorescein concentration was the response variable; the group (one or two drops), time (0–180 min), and group-by-time interaction were treated as fixed effects, and the animal and animal-by-group interaction were treated as random effects, using animal as block. After the model was fit, the fixed effects were tested, and comparisons between one vs. two drops were made for the following outcomes: (i) fluorescein concentration in tears at each time point, and (ii) percent of fluorescein remaining at each time point, using the baseline data of one drop for both groups in order to account for the different volumes instilled in both eyes. The R software was also used to calculate the area under the concentration-time curve (AUC), a parameter that was compared between groups (one vs. two drops) using the paired *t-*test. Differences among volume instilled in fluorescein periocular spillage and tear turnover rate were assessed with a one-way ANOVA, while the relationship between the volumetric capacity and the palpebral fissure length was assessed with the Pearson's correlation test. Statistical analyses were performed with SigmaPlot 14.0 (Systat software, Point Richmond, CA), and *P* < 0.05 were considered significant.

## Results

Data were normally distributed (*P* > 0.05), therefore results are presented as mean ± standard deviation (range).

### Volumetric Capacity of the Canine Palpebral Fissure

**Figure 3:**
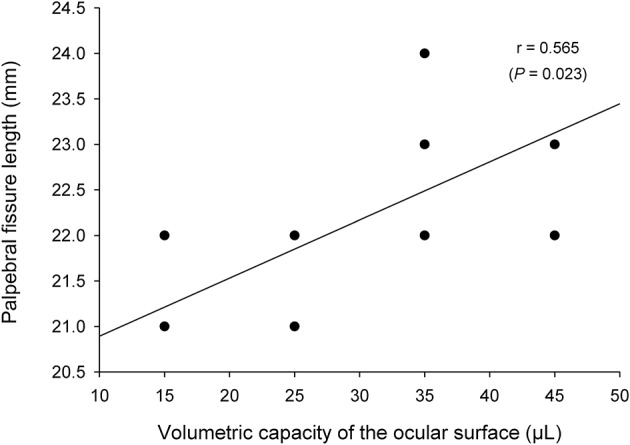
A positive association was found between the length and the volumetric capacity of the palpebral fissure (Pearson's correlation test).

Mean ± SD (range) volumetric capacity of the canine palpebral fissure was 31.3 ± 8.9 μl (15–45 μl). A moderate positive correlation (*r* = 0.57, *P* = 0.023) was found between the length (in mm) and the volumetric capacity (in μl) of the palpebral fissure (Figure 3). Further, mean palpebral fissure length and volumetric capacity were slightly larger in male dogs (22.5 mm and 35 μl) compared to female dogs (22 mm and 27.5 μl), although these differences were not statistically significant (*P* ≥ 0.090).

### Periocular Spillage of Instilled Solution

**Figure 4:**
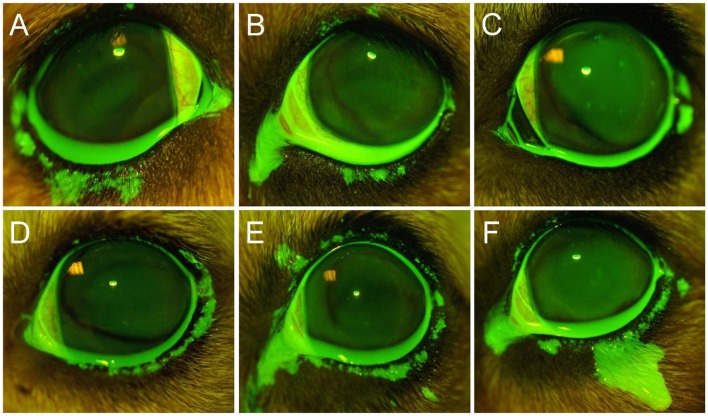
Representative ocular images following instillation of 60 μl **(A)**, 80 μl **(B)**, 40 μl **(C)**, or 100 μl **(D–F)** of 0.1% fluorescein solution in different Beagle dogs. Notice the periocular spillage that predominantly affects the lower eyelid **(A)**, medial canthus **(B)**, lateral canthus **(C)**, or multiple locations including the upper eyelid **(D–F)**.

**Figure 5:**
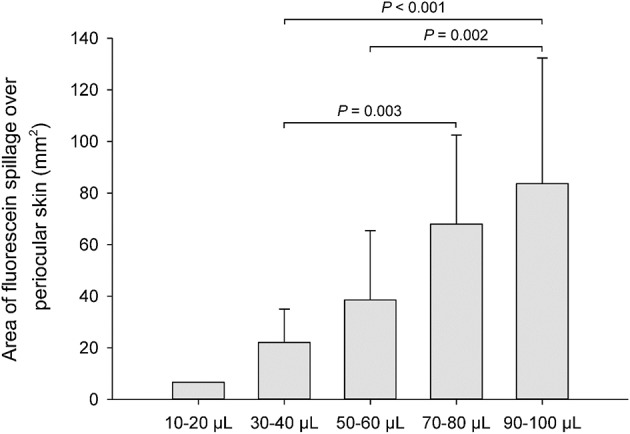
Bar chart depicting the mean area (+SD) of periocular spillage of 0.1% fluorescein solution, instilled at various volumes (10–100 μl) in 8 Beagle dogs (*n* = 16 eyes).

The lower eyelid represented the most common location (92%, Figures 1B, 2, 4A) covered by fluorescein spillage from the ocular surface, followed by the medial canthus (73%, Figure 4B), the lateral canthus (68%, Figure 4C), and the upper eyelid (32%, Figures 4E,F). Instillation of large volumes often resulted in excessive periocular spillage that covered multiple skin locations, although the amount and distribution of spillage varied within and between dogs; for instance, instillation of 100 μl of fluorescein onto the left eye of 3 different dogs resulted in either mild (Figure 4D) or pronounced (Figures 4E,F) periocular spillage. Overall, the area of periocular spillage increased as the volume of instilled solution increased (Figure 5), with statistical differences noted between 90 and 100 μl vs. 50 and 60 μl (*P* = 0.002), 90–100 μl vs. 30–40 μl (*P* < 0.001), and 70–80 μl vs. 30–40 μl (*P* = 0.003).

### Tear Turnover Rate

**Figure 6:**
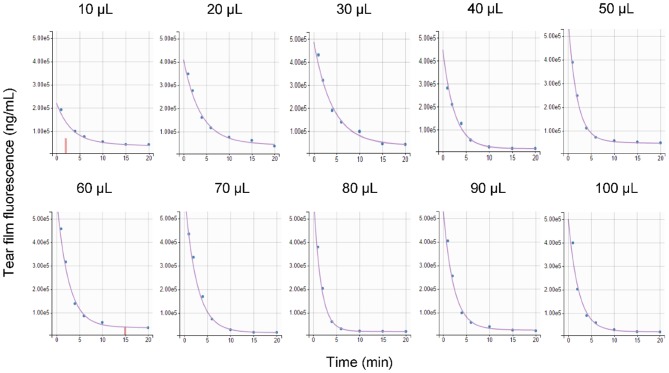
Comparison of predicted tear fluorescence over time (purple curve) with observed data (blue points) following topical instillation of 0.1% fluorescein solution (10–100 μl) in a representative Beagle dog. Censored data are shown as vertical red bars.

Parameter estimation was performed using the stochastic approximation expectation maximization algorithm for non-linear mixed-effects models implemented in the Monolix Suite, as previously described for analysis of canine pharmacokinetic data (23, 24). Standard goodness-of-fit diagnostics were used to assess the validity of the model, including visual predictive checks, individual predictions vs. observations, individual weighted residuals plotted against tear fluorescein concentrations, and simulations of fluorescein *vs*. time disposition from 500 Monte Carlo simulations (Supplementary Appendix). Using the final mathematical model, a visual inspection of individual fluorescein decay curves showed a tendency for a “steeper” initial slope (i.e., a faster tear drainage) with increasing volumes of instilled fluorescein, as seen in a representative animal that received 10–100 μl of topical solution (Figure 6). However, the average rTTR (20.2–30.5%/min) did not vary significantly among groups (*P* = 0.935), nor did the bTTR (1.1–1.4%/min, *P* = 0.988) observed a few minutes following fluorescein instillation (Table 1).

**Table 1:** Mean ± standard deviation of reflex tear turnover rate (rTTR) and basal tear turnover rate (bTTR) in 8 Beagle dogs following topical instillation of 10–100 μl of 0.1% fluorescein solution in each eye.

**Volume (μL)**	**10**	**20**	**30**	**40**	**50**	**60**	**70**	**80**	**90**	**100**	***P*-value**
rTTR (%/min)	20.6 ± 14.5	20.2 ± 11.4	22.7 ± 17.1	24.3 ± 12.0	30.3 ± 23.3	22.3 ± 13.0	24.6 ± 13.2	24.6 ± 18.4	30.5 ± 22.1	22.2 ± 11.6	0.935
bTTR (%/min)	1.4 ± 0.4	1.3 ± 0.4	1.3 ± 0.5	1.1 ± 0.5	1.2 ± 0.6	1.2 ± 0.6	1.1 ± 0.6	1.2 ± 0.5	1.1 ± 0.6	1.2 ± 0.6	0.988

*P-values depict the results of one-way ANOVA testing*.

### Tear Film Fluorescein Concentrations Following One vs. Two Drops

**Figure 7:**
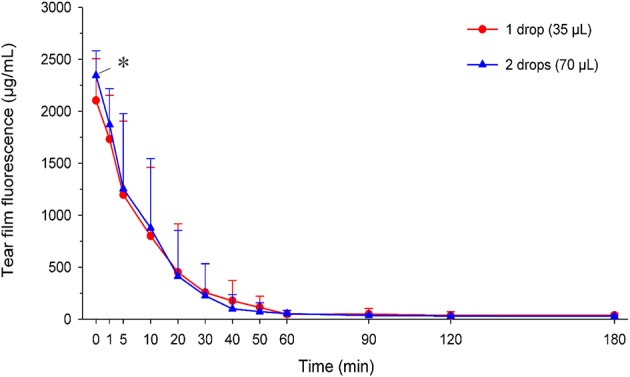
Scatter plot depicting the mean + SD of tear film fluorescence over time in canine eyes receiving either one drop (35 μl; red circles) or two drops (70 μl; blue triangles) of 1% fluorescein solution. Differences in tear fluorescence were noted at t = 0 min (*P* = 0.046) but no other time points (*P* ≥ 0.163).

Tear film fluorescein concentrations in eyes receiving one vs. two drops are depicted in Figure 7. Immediately following instillation of fluorescein (*t* = 0 min), tear film concentrations were significantly higher (*P* = 0.046) in eyes receiving two drops (2,345 ± 237 μg/ml) compared to one drop (2,104 ± 403 μg/ml). However, no statistical differences in fluorescein concentrations were noted at *t* = 1 min (*P* = 0.163) or any subsequent time points (*P* ≥ 0.293). In fact, the overall exposure of the ocular surface to fluorescein (AUC of fluorescein concentration-time curve) was slightly higher in eyes receiving one drop (30,513 ± 21,530 μg^*^min/ml) compared to two drops (28,975 ± 17,410 μg^*^min/ml). However, this difference was not statistically significant (*P* = 0.742), and the overall effect of volume instilled on tear film fluorescein was non-significant (*P* = 0.619) when taking “time” into account in the model.

**Figure 8:**
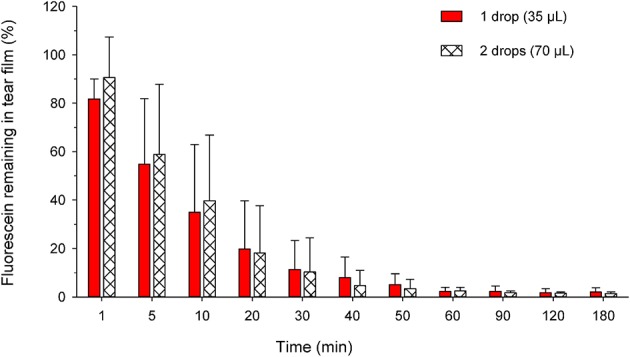
Bar chart depicting the mean + SD of residual tear film fluorescence at each time point in canine eyes receiving either one drop (35 μl; plain red bars) or two drops (70 μl; hatched white bars) of 1% fluorescein solution. For standardization, the residual fluorescence in both groups was compared to the tear fluorescence obtained at t = 0 min in eyes receiving a single drop of fluorescein. No statistical differences were noted between both groups at any time point (*P* ≥ 0.220).

The percentage of solution retained on the ocular surface following one vs. two drops is summarized in Figure 8. At *t* = 1 min, the percent retained was higher in the eyes receiving two drops (90.6 ± 16.7%) compared to one drop (81.8 ± 8.2%), a difference that approached statistical significance (*P* = 0.071). However, no significant differences were noted for any other time point (*P* ≥ 0.220) or in the overall effect of volume instilled on the percentage of solution retained (*P* = 0.731) when the variable “time” was taken into account.

## Discussion

The present study supports the use of a single eyedrop in dogs, whether used therapeutically in canine patients with ocular disease or experimentally in canine models of translational research (25, 26). A second drop achieved higher tear film concentrations immediately after topical administration (*t* = 0 min), a finding that is partly explained by a lower dilution effect for two drops (1.9-fold) than one drop (2.9-fold) from the tear fluid present on the canine ocular surface (~65 μl) (17). However, the benefit of instilling two drops was short-lived (<1 min) and unlikely to be clinically important, although the present study focuses on fluorescein and cannot be directly extrapolated to ophthalmic drugs such as antibiotics, corticosteroids or anti-glaucoma medications. A second drop is wasted from an economic perspective and can potentially exacerbate local and/or systemic adverse effects by overflow on the periocular skin and drainage through the nasolacrimal duct, respectively. The latter was not evaluated herein as fluorescein is non-biologically active, albeit previous studies have shown that overwhelming the lacrimal system can increase the amount of drug that reaches the blood *via* the naso-buccal mucosa (27). In sum, the kinetic profile of fluorescein in tears was not superior with two drops vs. one drop, a finding that is often explained by an accelerated lacrimal drainage with increasing volume in both rabbits (13) and humans (3). However, this explanation is not valid in dogs as the rate of lacrimal drainage did not change significantly in our canine subjects despite a 10-fold increase in instilled volume (10–100 μl); rather, the present study shows that excessive periocular spillage is the main culprit limiting the benefit of using two topical drops in canine ophthalmology. The amount of solution that overflowed on the periocular skin was greater with larger volumes of instilled solution and primarily affected the lower eyelid, medial canthus, and lateral canthus. Such spillage can participate to local adverse effects, such as *Malassezia* sp. overgrowth in dogs receiving topical medications (28), or skin hyperpigmentation and lengthening of eyelashes in humans receiving topical prostaglandin analogs (29).

The volumetric capacity of the canine palpebral fissure is 31.3 μl. This value is somewhat similar to the volumetric capacity in humans (25–30 μl) (1, 14), and approximates the average volume of a single eyedrop of commercial preparations (~35 μl) (19, 20). As such, a single eyedrop is deemed sufficient in dogs and humans because their ocular surface is unable to accommodate volumes larger than ~30 μl, yet therapy with a single drop is relatively inefficient in both species given the short precorneal residence time and low ocular bioavailability (30). Several strategies can be implemented to enhance the benefits of eyedrop administration, including: (i) Eyelid closure and/or nasolacrimal punctal occlusion for several minutes following topical instillation (6, 31, 32); (ii) Higher drug concentration—Walters et al. showed that topical 1.5% levofloxacin in humans achieved tear concentrations that were 3–10 times higher than those seen with 0.3% ofloxacin at multiple time points over 24 h (11); (iii) Higher solution viscosity and/or use of mucoadhesive polymers (15, 33); (iv) Administration of a second drop ≥1 min apart from the first drop—Herring et al. showed that administration of two drops (1-min apart) of 0.5% proparacaine in dogs achieved significantly greater and longer anesthetic effect compared to eyes that received a single drop (34); and (v) Use of volumes smaller than the average commercial drop size (16, 35).

Strategies to improve ocular drug delivery should ideally be investigated in each species separately, as direct extrapolation between species is hindered by differences in ocular anatomy and physiological parameters such as blink and tear turnover rates (13, 14, 17, 36). Rabbits, for instance, have a much slower blink rate (3–6 blinks/h) (37, 38) and a slower tear drainage (7%/min) (13) compared to humans (17 blinks/min and 10–20%/min, respectively) (18, 39) as well as a different expression of mucins on the ocular surface that could affect the retention of mucoadhesive polymers (40). These differences explain why an instilled eyedrop is partially lost (20–30%) due to reflex blinking and periocular spillage in humans, but not in rabbits (15, 37), or why a solution's viscosity has a great impact on precorneal retention and drug ocular bioavailability in humans, but not in rabbits (15, 41). In a study from over four decades ago, it was recognized that “considerable reservations may be felt about comparing results from rabbits with those from humans because of the differences between the physiology of tear flow and mixing and general anatomy,” yet “the rabbit is the principal experimental animal in ophthalmology, so comparisons are needed” (42). Since then, rabbits continued to be the “species of choice” for ophthalmic studies given their availability and easy handling, yet the present study shows that dogs likely represent a more relevant model for translational research. Indeed, dogs and humans share many similarities that are relevant to ophthalmic drug delivery, although important differences exist (e.g., tear volume) (17) that should be accounted for in comparative studies. The similarities include the blink rate (14.2 vs. 17 blinks/min) (39, 43), basal TTR (12.2 10–20%/min) (17, 18), reflex TTR following eyedrop instillation (20–30 vs. 30%/min, respectively) (44), volumetric capacity of the palpebral fissure (31.3 vs. 25–30 μl) (1, 14), and periocular spillage of excess solution. The aforementioned similarities justify the use of dogs as a translational model in ophthalmic research, especially given the presence of spontaneous canine diseases that closely resemble human conditions including keratoconjunctivitis sicca (45, 46), herpetic keratitis (47), and neurotrophic keratopathy (48).

The main limitation of the study is the use of dogs from a single breed (Beagles), all being ophthalmoscopically healthy and relatively young (3–3.5 years). The tear film pharmacokinetics of two drops may be different in a larger canine breed, presumably due to differences in volumetric capacity and/or tear drainage, as shown in German Shepherd dogs using the fluorescein clearance test (20). Similarly, the ocular surface of older dogs may accommodate a larger volume due to laxity in the eyelids, and the instilled solution may be retained for longer durations due to reductions in tear volume, reflex tearing, and tear turnover rate (49, 50). In addition, the present findings do not fully represent the physiology of eyes with ocular surface disease, in which chemosis can reduce the volumetric capacity of the palpebral fissure (51), inflammation can affect tear drainage (52), and ocular absorption (53), and excessive tearing from ocular irritation can further dilute the administered solution (54). In particular, patients with inflamed nasolacrimal duct (dacryocystitis) may actually benefit from instillation of a second drop, as greater nasolacrimal drainage would theoretically be beneficial in such cases. A second limitation of the study is related to the use of sodium fluorescein as a marker for tear film kinetics. Fluorescein was shown to overestimate tear turnover in human subjects, as a portion of instilled fluorescein can be lost by conjunctival permeation and not nasolacrimal drainage (55). However, a common alternative described by other investigators (i.e., gamma scintigraphy) (56) is not applicable to dogs, in whom the general anesthesia required to hold still for the procedure would negatively impact the tear film dynamics.

The present study on drop size and tear film pharmacokinetics can be summarized as follows. Instillation of two drops provided tear film fluorescein concentrations that were higher than one drop at baseline, due to lower dilution effect from tears, although the benefits were short-lived (<1 min) and not clinically important. The kinetic profile of fluorescein in tear film was not superior in eyes receiving two drops vs. one drop as determined by the residual tear fluorescence at various time points and the overall exposure of the ocular surface (AUC) to the solution instilled. Therefore, a single standard size drop is sufficient for topical administration in dogs, a finding supported by the volumetric capacity of the canine palpebral fissure (31.3 μl). Any excess is lost predominantly by spillage over the periocular skin as well as accelerated nasolacrimal drainage.

## Data Availability Statement

The datasets generated for this study are available on request to the corresponding author.

## Ethics Statement

The animal study was reviewed and approved by Institutional animal care and use committee of Iowa State University.

## Author Contributions

LS conceptualized and designed the study in consultation with RA and JM. LS, NK, and AR performed the experiments. LS and JM analyzed the data. All authors wrote the manuscript.

### Conflict of Interest

The authors declare that the research was conducted in the absence of any commercial or financial relationships that could be construed as a potential conflict of interest.
