# Supplementary material for: Kinetics of Fluorescein in Tear Film After Eye Drop Instillation in Beagle Dogs: Does Size Really Matter?
Source: Front Vet Sci. 2019 Dec 19;6:457. doi: 10.3389/fvets.2019.00457 (PMC6930880; doi:10.3389/fvets.2019.00457)
Supplement: Supplementary file 1 [file Data_Sheet_1.PDF]

Supplementary Appendix

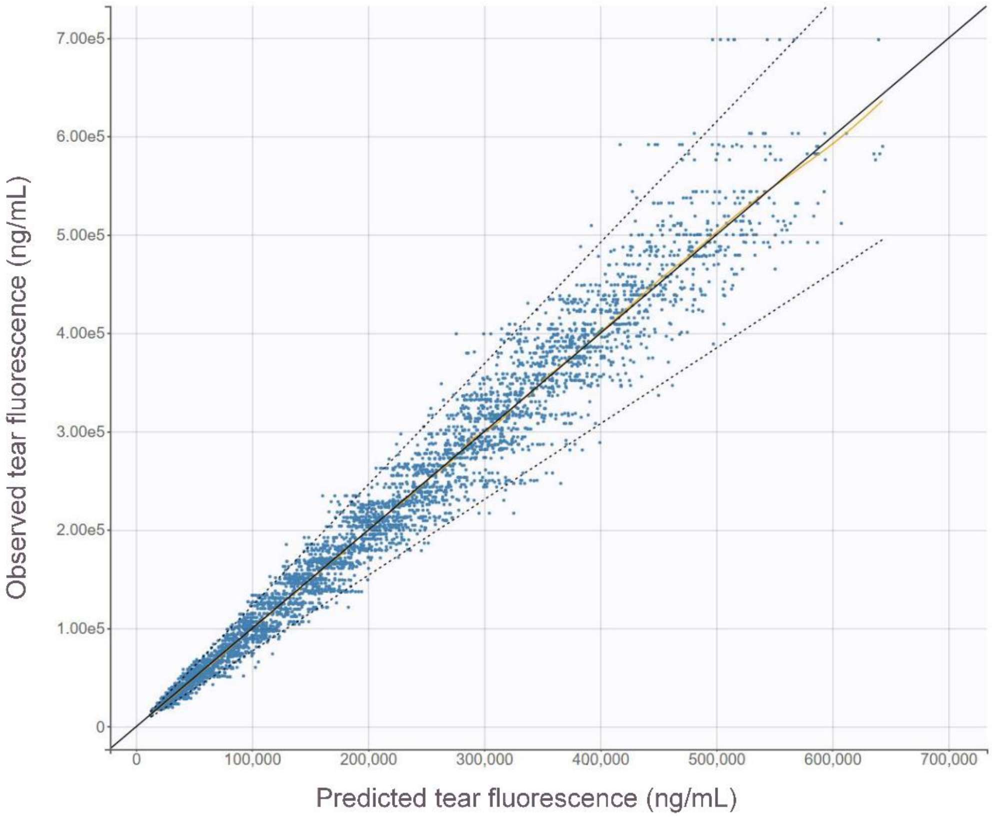

Supplementary Appendix Figure 1. Individual predictions versus observations (log10) for the fluorophotometry data in 8 dogs (16 eyes) receiving 10-100 µL of 0.1% fluorescein solution. The solid black line represents the identity line; the regression line is portrayed in light green color.

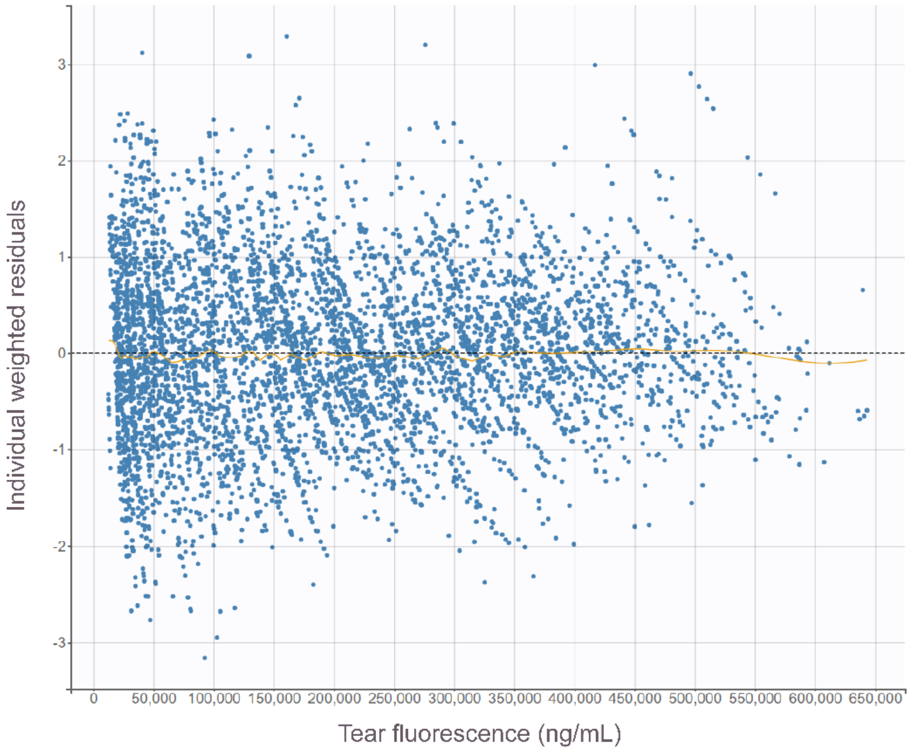

Supplementary Appendix Figure 2. IWRES plotted against the tear fluorescein concentrations in 8 dogs (16 eyes) receiving 10-100 µL of 0.1% fluorescein solution. The orange line represents the spline (loess regression). IWRES, individual weighted residuals.

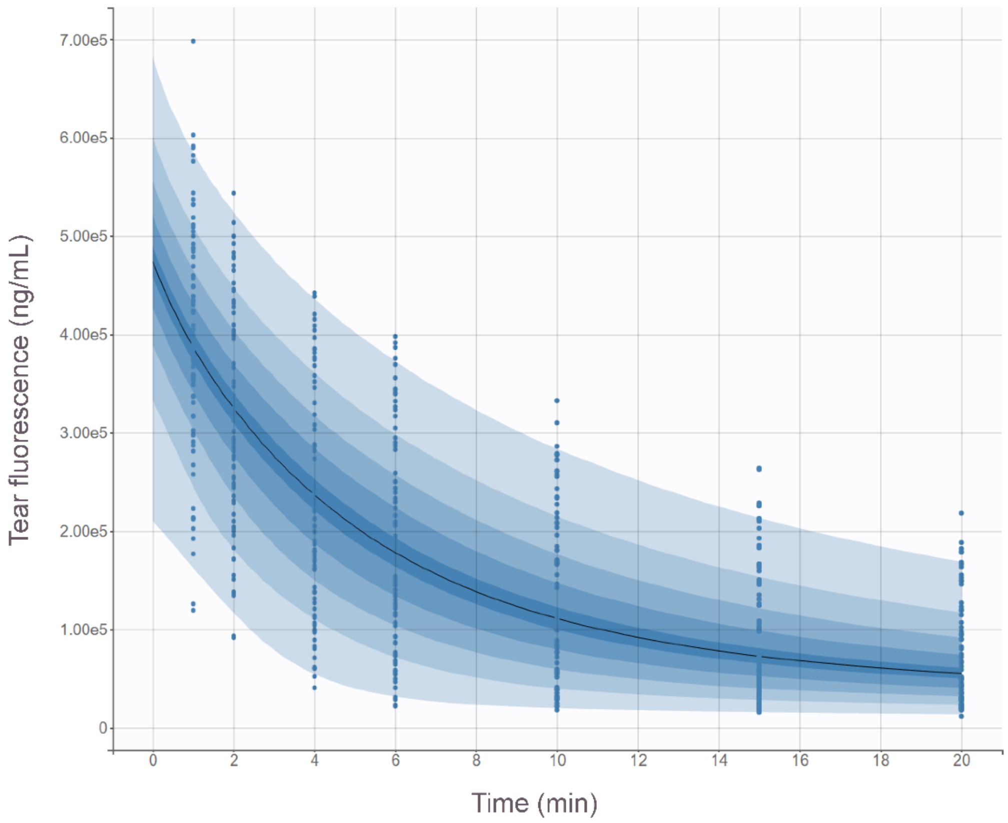

Supplementary Appendix Figure 3. Simulations of fluorescein vs. time disposition from 500 Monte Carlo simulations using final parameter estimates from the NLME model. Predictions derived from the 5th to the 95th percentile of the model simulations were able to reproduce the variability in the observed data from the original population dogs. NLME, nonlinear mixed effects.
